# Early Malnutrition Risk Detection for Quality Patient Care: An Analysis of Nutrition Risk Screening Tools in Kenyan Referral Hospitals

**DOI:** 10.3390/healthcare13233001

**Published:** 2025-11-21

**Authors:** Mahat Mohamed, Sophie Ochola

**Affiliations:** Department of Foods, Nutrition & Dietetics, The School of Health Sciences, Kenyatta University, Nairobi P.O. Box 43844-00100, Kenya

**Keywords:** nutrition risk screening tools, hospital malnutrition risk, validity of nutrition screening tools

## Abstract

**Highlights:**

**What are the main findings?**

- Compared the performance of three validated nutrition risk screening tools in Kenyan referral hospitals.
- NRS-2002 identified the highest prevalence of malnutrition risk, while BMI reported the lowest.
- MUST demonstrated the best overall diagnostic performance, with the highest AUC value (0.82).
- All tools showed high sensitivity but low specificity and limited agreement with BMI criteria.

**What are the implications of the main findings?**

- Adopting MUST as the standard screening tool can improve early detection and management of hospital malnutrition in Kenyan referral hospitals.
- Context-specific validation of nutrition screening tools is essential to ensure accurate, evidence-based nutritional care in low- and middle-income settings.

**Abstract:**

**Background & Objectives**: Malnutrition is a prevalent condition linked to increased morbidity and mortality among hospitalized adult patients. This study compared three nutrition risk screening tools—Nutritional Risk Screening 2002 (NRS-2002), Malnutrition Universal Screening Tool (MUST), and Malnutrition Screening Tool (MST)—to determine the most effective method for early malnutrition risk detection among adult inpatients across Kenyatta National Hospital, Moi Teaching and Referral Hospital, and Kisii National Teaching and Referral Hospital in Kenya. **Materials and Methods**: A cross-sectional analytical design involved 420 adult inpatients screened within 24 h of admission, using body mass index (BMI) as the reference standard. **Results**: Among hospitalized adults in three Kenyan referral hospitals, participants were predominantly middle-aged (median = 35.5 years). NRS-2002 identified the highest malnutrition risk prevalence (85.7%), followed by MST (71.7%) and MUST (51.0%), whereas BMI classified only 23.8% as at risk. Both NRS-2002 and MUST showed perfect sensitivity (1.000) but MUST demonstrated higher specificity (0.65) and superior diagnostic accuracy (AUC = 0.82). Agreement with BMI was low across all tools, though MUST showed the best overall consistency and balance between sensitivity and specificity. **Conclusions**: The study revealed significant variation in malnutrition risk detection across NRS tools. While NRS-2002 was highly sensitive, it tended to overestimate risk. MUST demonstrated the best overall balance of sensitivity, specificity, and diagnostic accuracy. Adoption of MUST as the standard screening tool in Kenyan referral hospitals is recommended to enhance early malnutrition detection and improve patient care outcomes.

## 1. Introduction

Malnutrition is among the most prevalent comorbidities in hospitalized patients, affecting 20–50% of this population globally [1,2,3,4,5,6,7]. Malnutrition is considered as an independent risk factor for morbidity and mortality, imposing substantial burdens on healthcare systems [8,9,10,11,12,13]. Globally, at least one-third of hospitalized patients are malnourished at the time of admission, and if untreated, nearly two-thirds of these individuals experience further deterioration in nutritional status during hospitalization [14,15]. Hospitalization costs have been shown to increase by more than 60% among malnourished patients in international studies, largely due to prolonged length of stay and the added expense of managing nutrition-related complications; however, comparable data from Kenya remain limited [16,17]. Patients with malnutrition have a threefold greater risk of surgical site infection than well-nourished patients and are more likely to be readmitted within 15 days post-discharge [18,19,20,21]. A study by Prof. Renée Blaauw examining hospital malnutrition in the African continent, comparing South Africa, Kenya, and Ghana, demonstrated that malnutrition is highly prevalent and poses a serious threat to morbidity and mortality, contributing to prolonged hospitalization [22]. Similar patterns have been reported in Turkey [23].

Despite the high prevalence of malnutrition among patients prior to admission, the condition is frequently neglected, undiagnosed, and untreated, earning the designation “the skeleton in the hospital closet” [24,25,26,27,28]. Low-cost strategies for identifying at-risk patients through validated Nutrition Risk Screening (NRS) tools and delivering adequate nutrition support hold significant potential to improve outcomes and reduce healthcare expenditure [29,30,31,32]. However, although multiple validated NRS tools exist, no consensus has been established regarding the most effective tool for identifying hospitalized patients at risk of malnutrition [33,34]. The application of these screening instruments has not been specifically evaluated in Kenyan healthcare facilities, a context characterized by high mortality rates among hospitalized patients [33]. There remains a paucity of data on NRS practices in East Africa, including Kenya, where most research has focused on nutritional status assessment without addressing NRS. Globally, the NRS-2002, MUST, and MST are among the most widely validated tools for adult inpatient screening, recognized by the European Society for Clinical Nutrition and Metabolism (ESPEN) and the British Association for Parenteral and Enteral Nutrition (BAPEN). However, most validation studies have been conducted in high-income countries, where patient profiles, resource availability, and clinical workflows differ substantially from those in low- and middle-income countries (LMICs). In Kenyan referral hospitals, limited staff time, high patient turnover, and resource constraints make it critical to identify a tool that is not only accurate but also simple, quick, and compatible with existing ward practices. By validating these three practical screening tools within a Kenyan context, this study provides evidence to guide context-appropriate clinical nutrition workflows and optimize early detection of malnutrition in LMIC hospital settings.

This study aimed to compare the diagnostic accuracy of three validated nutrition risk screening tools—NRS-2002, MUST, and MST—among adult inpatients in Kenyan national referral hospitals

## 2. Materials and Methods

### 2.1. Study Design and Variables

This hospital-based, cross-sectional analytical study assessed nutritional risk among adult inpatients in three Kenyan National Referral Hospitals. Data were collected using three validated NRS tools: the NRS-2002, MUST, and MST. Independent variables representing malnutrition risk factors included recent unintentional weight loss, reduced food intake or appetite, and the presence of an acute disease condition. Dependent variables comprised the proportions of cases classified as at risk of malnutrition or normal according to each tool. Diagnostic performance was evaluated by calculating sensitivity, specificity, and receiver operating characteristic (ROC) curves. The European Society for Parenteral and Enteral Nutrition (ESPEN) diagnostic criterion for malnutrition, defined as body mass index (BMI) < 18.5, served as the reference standard [35].

### 2.2. Study Location and Target Population

The study was conducted in the medical and surgical wards of Kenyatta National Hospital (KNH), Moi Teaching and Referral Hospital (MTRH), and Kisii Teaching and Referral Hospital (KTRH). These public facilities provide specialized medical services nationwide, receive referrals from all regions, and function as research and training centres for healthcare professionals. The study population comprised adult patients aged 18–64 years who were admitted to the medical and surgical wards within 24 h of hospitalization.

### 2.3. Exclusion Criteria

Patients admitted for day cases, pregnant or breastfeeding females, individuals with moderate to severe edema, and bedridden patients were excluded to preserve the accuracy and validity of NRS. Patients hospitalized for <24 h were excluded because comprehensive clinical assessments are not conducted during brief admissions, and detailed nutritional screening is not feasible within such encounters. Females who were pregnant or breastfeeding were excluded due to physiological changes, including fluid retention, altered body composition, and weight gain related to pregnancy and lactation. These factors compromise the accuracy of anthropometric measurements and reduce the reliability of standard adult screening tools. Bedridden patients were excluded because measuring body weight and height accurately is often impractical. Additionally, prolonged immobility and pre-existing conditions, such as muscle wasting or fluid retention, may alter body composition and limit BMI validity. Patients with moderate to severe oedema experience fluid accumulation, artificially increasing body weight and potentially leading to underestimation of malnutrition risk when BMI is used. Individuals aged < 18 years were excluded, as paediatric patients require age-specific and developmentally appropriate nutritional screening instruments.

A total of 150 patients were excluded based on predefined criteria. Specifically, 40 patients were excluded due to being day case admissions, as they did not meet the minimum 24 h hospital stay required for comprehensive clinical and nutritional screening for further assessment. The excluded were as follows:

- Twenty-five women who were pregnant or breastfeeding were excluded.
- Twenty patients with moderate to severe oedema were excluded.
- Thirty bedridden patients.
- Twenty patients were excluded because their hospital stay was less than 24 h.
- Fifteen individuals under the age of 18 were excluded, as the study focused solely on adult patients and required the use of age-appropriate nutritional assessment tools.

**Figure 1:**
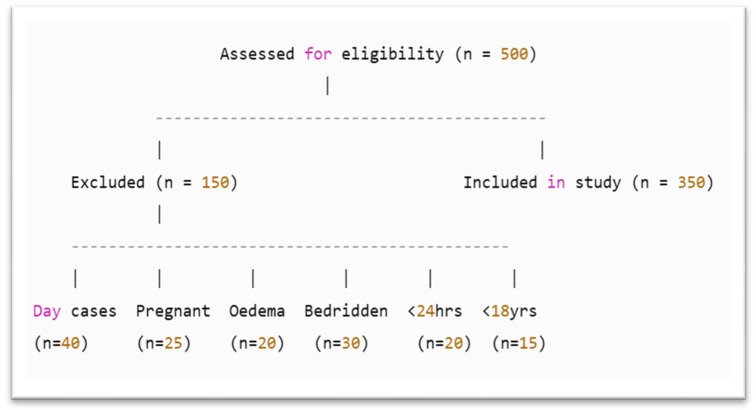
Flowchart of Participant Selection and Exclusion Criteria.

A total of 500 patients were assessed for eligibility (Figure 1).

### 2.4. Sample Size

Sample size was determined using the formula for a single population proportion [36], with a 95% confidence interval and 5% precision. A prevalence of malnutrition risk of 50% (0.5) was assumed to yield the maximum sample size, given the lack of prior studies in this context. An additional 5% was added to account for nonresponse. Finite population correction was applied to produce a sample proportional to the target population.

$$n=\frac{Z^{2}P\left(1-P\right)}{e^{2}}$$

where

n: Desired sample size;
e: Desired margin of error/desired precision (0.05);
Z: Standard normal deviate at 95% confidence level (1.96);
P: The prevalence of hospital malnutrition/nutritional risk in the target population was assumed to (0.5).

There was no previous national study with a hospital malnutrition rate comparable to that of the study.

$$=\frac{{\left(1.96\right)}^{2}\times 0.5\;(1-0.5)}{{\left(0.05\right)}^{2}}$$

= 385patients

A 5% nonresponse rate was incorporated into the final sample size, resulting in 420 patients equally allocated across three hospitals. Each facility enrolled 140 patients as follows: KNH (80 males, 60 females), MTRH (60 males, 80 females), and KTRH (96 males, 44 females).

### 2.5. Sampling Techniques

The three public National Referral and Teaching Hospitals were purposively selected as they were the only gazetted level 6 public teaching and referral hospitals in Kenya, excluding the National Spinal Injury Referral Hospital, which was omitted due to its specialization in spinal injury cases. In each hospital, stratified random sampling was used to select males and females from the medical and surgical wards. Subsequently, patients were chosen through simple random sampling using a Table of Random Numbers from the list of patients admitted in the preceding 24 h. Patients who provided consent were enrolled and screened within 24 h of admission. This process continued daily until the required sample size was reached.

### 2.6. Study Instruments and Data Collection

All participants were screened within 24 h of admission using the following NRS tools: NRS-2002, MUST, and MST. Relevant variables from each tool were recorded. Anthropometric data, including weight and height, were measured following standard procedures. Weight was recorded to the nearest 100 g using Seca 763 scales, and height was measured to the nearest 1 mm using a stadiometer. BMI was calculated using the formula BMI = kg/m^2^. BMI served as the reference standard for evaluating the performance of the screening tools, based on the ESPEN diagnostic criteria [35]. NRS-2002, MUST, and MST were purposely selected because they are validated screening tools recommended by ESPEN and BAPEN for rapid identification of adult inpatients at risk of malnutrition. In contrast, the SGA and PG-SGA are diagnostic assessment instruments that evaluate established malnutrition rather than screening for risk, and were therefore excluded from the present analysis

### 2.7. Reference Standard for the Study

The study adopted BMI as the reference standard, applying the ESPEN diagnostic criterion for malnutrition, defined as BMI < 18.5 kg/m^2^. This method aligns with several previous studies that utilized the same standard [5,35,37,38,39]. Therefore, BMI was adopted as a feasible and standardized proxy reference while recognizing its limitations, and the need for future validation of screening tools against composite or functional criteria.

### 2.8. Data Quality Assurance

Research assistants were selected from qualified nurses and nutritionists/dietitians and underwent a 3-day intensive training covering objectives, procedures, and interviewing techniques. All NRS tools used had been previously validated. To ensure accuracy in anthropometric measurements, data quality checks were conducted, including digit preference score analysis for weight and height to detect rounding errors.

### 2.9. Data Analysis

Data were initially organized in Excel and later imported into SPSS version 22 and STATA for analysis. The diagnostic performance of NRS-2002, MUST, and MST in identifying malnutrition risk among adult inpatients was assessed. Evaluation metrics included sensitivity (proportion of malnourished individuals correctly identified), specificity (proportion of well-nourished individuals correctly identified), positive predictive value (PPV: proportion of patients with a positive screening result who were malnourished), and negative predictive value (NPV: proportion of patients with a negative screening result who were truly well-nourished) [12,29,40,41,42]–interpretation of the scores is illustrated in Table 1. Inter-rater reliability was measured using Cohen’s kappa statistics, which evaluates agreement between raters using BMI as the reference standard. ROC curves were used to assess tool performance. The area under the curve (AUC) was interpreted as follows:

(1) 0.90 ≤ AUC ≤ 1.00: Excellent performance;
(2) 0.80 ≤ AUC < 0.90: Good performance;
(3) 0.70 ≤ AUC < 0.80: Fair performance;
(4) 0.60 ≤ AUC < 0.70: Poor performance;
(5) 0.50 ≤ AUC < 0.60: Failure.

The ESPEN diagnostic criterion (BMI < 18.5) was used in previous studies [35]. Statistical significance was determined at α = 0.05, with *p*-values < 0.05 considered statistically significant (95% confidence intervals [CIs]).

#### Ethical Considerations

Approval of conducting the research was granted by the Kenyatta University Graduate School ref. no. H87/37116/2017, the three hospitals and approved by the National Commission for Science, Technology, and Innovation (NACOSTI). no. NACOSTI/P/21/10259. Ethical approval was issued by Kenyatta University Ethical Review Committee (KUERC) and Kenyatta National Hospital-University of Nairobi (KNH-UON) Ethical Research Committee.

## 3. Results

### 3.1. Characteristics of the Study Participants

**Figure 2:**
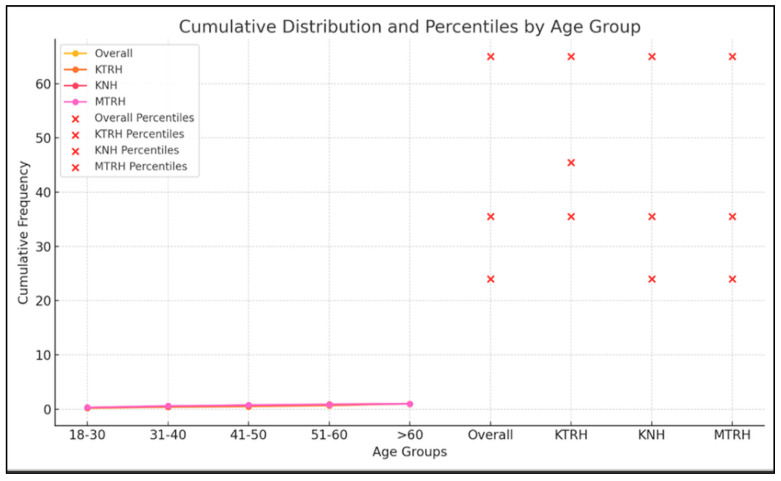
Cumulative Distribution & Percentiles by Age Group.

The study population was generally middle-aged. The 25th percentile for age was 24 years, the median (50th percentile) was 35.5 years, and the 95th percentile was 65 years, indicating that most participants were between 18 and 40 years (Figure 2). At KTRH, participants were older (median age: 45.5 years) compared to the overall population. In contrast, KNH and MTRH had younger distributions (median: 35.5 years), with similar upper age limits (95th percentile: 65 years). Overall, the three hospitals exhibited comparable demographic patterns, with a predominance of younger and middle-aged adults.

### 3.2. Prevalence of Hospital Malnutrition Risk

Malnutrition risk was assessed using NRS-2002, MUST, and MST, in addition to BMI classification (Table 2a).

- NRS-2002 identified the highest prevalence of malnutrition risk (85.7%);
- MST indicated 71.7%;
- MUST identified 51.0%;
- BMI classified only 23.8% as at risk.

Across hospitals, NRS-2002 consistently detected the highest prevalence (85–86%), followed by MST (61–83%), and MUST (49–54%). BMI consistently reported the lowest rates (22–30%). Pairwise comparisons using McNemar’s test revealed statistically significant differences between tools (*p* < 0.0001), confirming variability in risk detection accuracy (Table 2b).

### 3.3. Evaluation of the Effectiveness of Nutritional Screening Tools

The diagnostic performance of the three tools was assessed using sensitivity, specificity, PPV, NPV, and ROC curves (Table 3).

**Figure 3:**
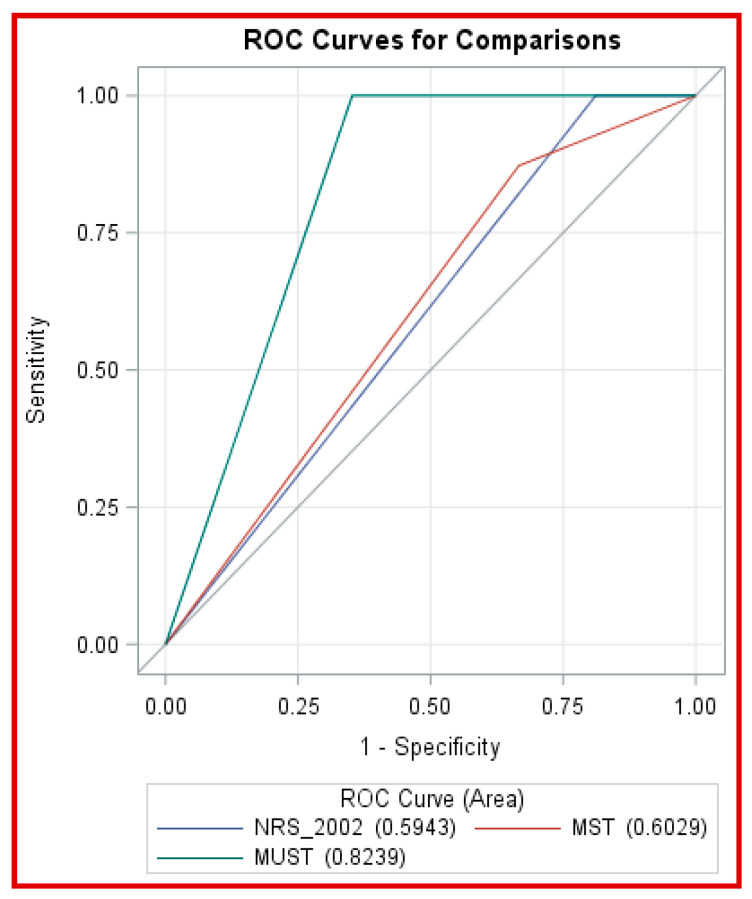
Receiver Operating Characteristic (ROC) Curves Comparing the Diagnostic Performance of NRS-2002, MUST, and MST. This figure provides a visual summary of the diagnostic accuracy results presented in Table 4, illustrating the relative sensitivity and specificity of each screening tool based on the area under the curve (AUC). Higher AUC values reflect better discriminative performance.

- Sensitivity: NRS-2002 and MUST both achieved perfect sensitivity (1.000), while MST showed slightly lower sensitivity (0.8725).
- Specificity: NRS-2002 had the lowest (0.1886), MST moderate (0.3333), and MUST the highest (0.6478).
- Predictive Values: Both NRS-2002 and MUST had perfect NPV (1.000), while MUST recorded the highest PPV (0.4766).
- ROC Analysis: MUST achieved the largest AUC (0.8239), indicating superior discriminative ability. MST (0.6029) and NRS-2002 (0.5943) performed comparatively lower (Figure 3).

Overall, MUST demonstrated the best balance between sensitivity, specificity, and predictive accuracy.

**Table 4:** Agreement among the NRS tools and BMI based on k-statistics.

Tool	k-Statistics	*p*-Value	Interpret
NRS-2002	0.2115 (0.1061–0.3169)	0.0011	low
MUST	0.2539 (0.2008–0.3069)	<0.0001	low
MST	0.1254 (0.0290–0.1822)	<0.0001	low

### 3.4. ROC Contrast Estimation—Comparison of NRS-2002, MUST, and MST

ROC contrast estimation confirmed that MUST outperformed both NRS-2002 and MST in diagnosing malnutrition risk.

- No significant difference was found between NRS-2002 and MST (*p* = 0.6996).
- MUST vs. NRS-2002: positive contrast estimate (0.2296), indicating better diagnostic accuracy.
- MUST vs. MST: positive estimate (0.2210, *p* < 0.05), showing significantly superior performance.

These results further confirmed MUST’s higher AUC (0.8239) compared to MST (0.6029) and NRS-2002 (0.5943), reflecting its superior diagnostic capacity (Figure 3, Table 5).

### 3.5. Agreement Between NRS Tools and BMI

Kappa statistics showed poor agreement between the screening tools and BMI classification (Table 4 and Table 6).

- NRS-2002: κ = 0.2115 (*p* = 0.001);
- MUST: κ = 0.2539 (*p* < 0.0001);
- MST: κ = 0.1254 (*p* < 0.001).

Similarly, inter-tool agreement was low to slight:

- NRS-2002 vs. MUST: κ = 0.2269;
- MUST vs. MST: κ = 0.2483;
- MST vs. NRS-2002: κ = 0.2269.

These findings indicate limited consistency among tools and between the screening instruments and BMI, reinforcing the need for tool selection suited to specific clinical contexts.

## 4. Discussion

This study revealed substantial disparities in the prevalence of malnutrition risk as assessed by three NRS tools. The reference criterion, (BMI), identified malnutrition in the smallest proportion of participants (23.8%). In contrast, the NRS-2002, MUST, and MST detected malnutrition risk in 85.7%, 51%, and 71.7% of participants, respectively. The BMI reference standard demonstrated the lowest prevalence and posed the least risk of overestimation. These findings indicate notable variability in the tools’ performance. Moreover, the prevalence of malnutrition varies across tools, populations, and clinical settings [23,45].

NRS-2002 identified the highest malnutrition risk, whereas BMI revealed the lowest. Similar studies have reported high nutritional risk in diverse populations using different screening tools [35,46,47,48,49]. Conversely, other research has described lower prevalence rates, underscoring the inconsistency of tool performance across demographic groups and care settings [50]. NRS-2002 has been shown to frequently overestimate malnutrition risk among hospitalized patients [51,52]. Despite similarities between NRS-2002 and MUST, their differing evaluation criteria likely explain the variation in prevalence, as observed in prior studies [53]. Although praised for its sensitivity, NRS-2002’s tendency to overestimate risk can result in unnecessary nutritional interventions, straining hospital resources and affecting patient care. Conversely, the BMI reference standard may fail to identify many patients at risk, potentially delaying intervention and leading to underestimation of malnutrition.

The absence of consensus on an ideal NRS tool remains a barrier to standardized nutritional care. The ESPEN strongly recommends NRS-2002 based on its content validity, strength, and capacity to accurately diagnose and predict nutritional risk [7,54]. Despite concerns about overestimation, ESPEN endorses the NRS-2002; however, its application across diverse clinical environments remains contentious. The lack of a universally accepted screening tool is further reflected in inconsistent findings across studies. Some research reports high nutritional risk prevalence with various NRS tools [46,47,48,49]. This discrepancy likely arises from differences in population characteristics, clinical contexts, and tool-specific criteria. For example, MUST and MST demonstrated differing prevalence rates, highlighting their distinct methodologies and evaluation standards [55].

Among the three tools, MUST exhibited the best overall performance, with high sensitivity, specificity, NPV, and the largest AUC. Although NRS-2002 achieved perfect sensitivity and NPV, it had low specificity and PPV, contributing to a higher rate of false positives. While widely implemented, NRS-2002 has been criticized for poor specificity [18,56]. MST demonstrated moderate sensitivity but had limitations in specificity and predictive accuracy. These findings suggest that although all three tools can identify malnutrition risk, their reliability varies, with MUST demonstrating the most balanced and effective performance in this analysis.

This study found that NRS-2002 and MUST showed slight agreement, indicating limited consistency in patient classification. In contrast, MUST exhibited fair agreement, suggesting better reliability compared to the other tools. Agreement between MUST and MST, and between MST and NRS-2002, remained slight, demonstrating limited concordance among these pairs. The fair agreement with MUST underscores its potential as a more dependable screening instrument. This inconsistency aligns with prior research [52,56]. Notably, the agreement between NRS-2002 and MUST in the present study corresponds to findings by Bellanti et al. [57]. In contrast, other investigations, including Neelemaat et al. [34], have reported higher specificity and sensitivity, possibly due to employing different reference standards. Misclassification remains a concern with NRS tools; earlier studies documented misclassification rates of 38% for NRS-2002 and 18% for MUST [17,18]. Overall, MUST demonstrated superior diagnostic accuracy in identifying patients at nutritional risk compared with NRS-2002 and MST. All three tools exhibited high NPVs, effectively identifying patients not at risk of malnutrition. However, low PPVs indicated their limitations in correctly classifying patients who were malnourished or at risk.

The agreement between the nutritional screening tools and BMI was evaluated using Cohen’s κ coefficient. NRS-2002 and MST demonstrated fair agreement with BMI, whereas MST exhibited no intra-researcher agreement. Significant statistical differences were identified between the tools and BMI (*p* < 0.05). Agreement strength was fair between MST and NRS-2002 and between NRS-2002 and MUST, while it was moderate between MUST and MST. The AUC analysis for predicting malnutrition risk revealed no significant difference between NRS-2002 and MST (*p* = 0.1489) but significant differences between NRS-2002 vs. MUST vs. MST (*p* < 0.05). These findings align with previous studies indicating a lack of consensus on the optimal NRS tool [23,45,58,59,60]. Bellanti et al. [57] reported that MUST and NRS-2002 exhibit higher specificity, especially in older adults. While the statistical comparisons among screening tools revealed some differences in diagnostic accuracy, not all statistically significant contrasts may translate into meaningful clinical improvement. Therefore, selection of a screening tool should prioritize practicality, consistency, and applicability within local hospital workflows rather than focusing solely on small statistical variations.

ROC analysis confirmed that MUST outperformed both NRS-2002 and MST in overall diagnostic accuracy. While all three tools demonstrated high negative predictive values, MUST provides the best balance between sensitivity and specificity, making it the most reliable for identifying patients truly at risk of malnutrition without excessive false positives. These findings highlight the tool’s suitability for routine use in hospital settings where rapid and accurate screening is essential [55]. The observed area under the curve (AUC) of 0.82 for MUST indicates a “good” level of discrimination, signifying that the tool correctly distinguishes patients at risk of malnutrition from those who are well-nourished approximately 82% of the time. In clinical terms, this reflects a high capacity for early identification of at-risk patients with minimal misclassification. However, while the differences in AUC values among the tools were statistically significant, their practical or clinical implications may be modest. For instance, a small numerical improvement in AUC does not necessarily translate into a substantial difference in patient outcomes. Therefore, interpretation should consider both statistical validity and real-world clinical relevance when selecting screening tools for routine hospital use.

The study concluded that none of the three nutritional screening tools exhibited high statistical efficacy for predicting malnutrition because of limited validity. However, MUST demonstrated superior criterion validity compared to NRS-2002 and MST. Although ESPEN guidelines recommend NRS-2002 for hospital use, these results suggest that MUST may be more effective for malnutrition screening. The findings offer valuable insights for selecting appropriate tools to better identify and manage adult inpatients at risk of malnutrition. Further research is necessary to validate these findings across different populations and settings. The study’s specific focus on Kenyan national teaching and referral hospitals restricts generalizability, requiring additional research in other regions with similar contexts [59].

This study had several limitations. First, the analysis relied on BMI < 18.5 kg/m^2^ as the reference standard, which, while practical, may not fully capture malnutrition risk among patients with edema, obesity, or muscle wasting/sarcopenia. Second, biochemical and inflammatory markers such as serum albumin and C-reactive protein (CRP) were not included, which could have provided a more comprehensive assessment of nutritional and inflammatory status. Third, inter-rater reliability among assessors was not evaluated, which may have introduced variation in tool performance. Additionally, bedridden and edematous patients were excluded due to measurement constraints, potentially limiting the generalizability of the results. Finally, the study was conducted in only three national referral hospitals, so the findings may not be representative of lower-level health facilities thus future validation should extend to county and district hospitals to confirm applicability in varied healthcare levels. Finally, the present study focused on three widely validated and practical screening tools: NRS-2002, MUST, and MST purposely selected for their applicability in routine hospital care and their validation for use in hospital settings. Therefore, the findings may not be generalizable to all existing nutrition screening instruments.

Future studies should address these limitations by incorporating functional, biochemical, and multi-site validation approaches to enhance the applicability of results across different hospital contexts. Given its diagnostic balance, simplicity, and alignment with hospital workflows, MUST should be integrated into routine inpatient screening protocols. Its adoption—combined with periodic re-screening and clinician judgment—can strengthen early identification of malnutrition and improve patient outcomes.

## 5. Conclusions

This study compared three nutrition risk screening tools—NRS-2002, MUST, and MST—to identify the most effective instrument for early malnutrition risk detection among adult inpatients in Kenyan referral hospitals. The findings show that although all three tools demonstrated high sensitivity, their specificity and agreement with BMI varied considerably. MUST achieved the most balanced diagnostic accuracy and is therefore recommended as the preferred screening tool in similar hospital settings. Integrating MUST into hospital admission protocols, supported by periodic re-screening and clinician judgment, could strengthen early identification and management of malnutrition, ultimately improving patient outcomes. Further validation in other healthcare levels across Kenya is recommended to enhance generalizability. Although widely accepted as a reference standard, BMI might not be an ideal benchmark for NRS, as factors such as edema, hydration status, lean body mass, and adipose tissue can influence BMI values [61,62]. The largest discrepancy was observed between the NRS-2002 and MUST, suggesting that MUST may fail to detect cases identified by the NRS-2002. While the NRS-2002 is the most sensitive, it is prone to overestimating malnutrition risk, which may strain hospital resources. While overestimation of malnutrition risk can burden healthcare resources through unnecessary interventions, underestimation may be clinically more detrimental, as patients genuinely at risk may not receive prompt nutritional support. Such false negatives can contribute to worsening nutritional decline, delayed wound healing, and extended hospitalization. Therefore, despite its slightly higher specificity, MUST should be accompanied by periodic re-screening and clinical judgment to ensure that no patient in need of nutrition care is overlooked. It’s noteworthy that false negatives may lead to missed nutritional interventions and delayed recovery and that low kappa values indicate variability across tools, underscoring the need for context-specific screening protocols and periodic reassessment.

These findings underscore the substantial variability in screening outcomes and indicate that tool selection directly affects the identification of at-risk patients. Selecting an appropriate NRS instrument suited to the clinical context and patient population is essential for accurate detection. Despite concerns regarding overestimation, the ESPEN strongly endorses NRS-2002 [7,63]. Although simpler, MST demonstrated limited accuracy in this setting. Variations in malnutrition prevalence across the tools may arise from their distinct methodologies, evaluation criteria, and differences in population demographics and clinical environments [43,55]. The results revealed significant differences in malnutrition risk identification. In this study, MUST showed the most favorable overall performance, consistent with other research findings [63]. Although all tools demonstrated high sensitivity and NPV, their low specificity and limited correlation with BMI raise concerns about their ability to accurately identify malnutrition. While NRS-2002 is sensitive, it may overestimate risk, whereas MUST provides a more balanced assessment. Given its diagnostic balance, simplicity, and compatibility with hospital workflows, MUST should be adopted as the standard nutrition risk screening tool in Kenyan referral hospitals. Incorporating MUST into admission protocols—alongside periodic re-screening and clinician judgment—will strengthen early malnutrition identification and improve patient outcomes.

The study recommends the following: (a) Adopt MUST as the primary screening tool for adult inpatients in Kenyan referral hospitals. (b) Develop implementation guidelines and protocols tailored to Kenyan clinical settings, integrating MUST into routine hospital admission processes. (c) Establish national nutrition screening policies reflecting evidence-based practices while allowing flexibility for context-driven tool selection to ensure timely and accurate risk detection.

These findings highlight the importance of context-specific, evidence-based nutritional screening approaches. By adopting MUST and supporting its integration into clinical workflows, healthcare systems in Kenya can strengthen nutritional care. The significant disparities among instruments underscore the absence of a universally optimal screening tool and reinforce the need for context-appropriate selection. Further research evaluating NRS tools across diverse patient demographics and clinical contexts in Kenya and other low- and middle-income countries is necessary to validate these results and advance evidence-based practice.

## Figures and Tables

**Table 1:** Interpretation of screening tool validity and reliability.

Validity	High	Moderate	Low
Sensitivity	≥90%	70–89%	<70%
Specificity	≥90%	70–89%	<70%
Negative predictive value	≥90%	70–89%	<70%
Agreement (Cohens kappa)	≥0.8	0.6–0.79	<0.6

Source: Indicators [34,43,44].

**Table 2:** (**a**) Prevalence of hospital malnutrition risk estimated by different NRS tools and by ESPEN (BMI) criteria. (**b**) Pairwise comparison between NRS tools in determining malnutrition risk.

(a)					
Tool	Category	Proportions of Normal and at-Risk Cases per Facility			
		Overall, N = 420	KTRH, N = 140	KNH, N = 140	MTRH, N = 140
NRS-2002	Normal	60 (14.3)	19 (13.6)	20 (14.3)	21 (15.0)
	At-risk	360 (85.7)	121 (86.4)	120 (85.7)	119 (85.0)
MUST	Normal	206 (49)	64 (45.7)	71 (50.7)	71 (50.7)
	At-risk	214 (51)	76 (54.3)	69 (49.3)	69 (49.3)
MST	Normal	119 (28.3)	54 (38.6)	41 (29.3)	24 (17.1)
	At-risk	301 (71.7)	86 (61.4)	99 (70.7)	116 (82.9)
BMI	Normal	320 (76.2)	109 (77.9)	101 (72.1)	108 (77.1)
	At-risk	100 (23.8)	31 (22.1)	39 (29.9)	32 (22.9)
(**b**)					
**Comparison**			***p*** **-Value**		
NRS vs. MST			<0.0001		
NRS vs. MST			<0.0001		
MUST vs. MST			<0.0001		

**Table 3:** Performance of NRS tools against BMI.

	NRS-2002	MUST	MST
Se	1.0000 (0.9645–1.0000)	1.0000 (0.9645–1.0000)	0.8725 (0.8078–0.9373)
Sp	0.1886 (0.1457–0.2317)	0.6478 (0.5925–0.7003)	0.3333 (0.2817–0.3881)
NPV	1.0000 (0.9404–1.0000)	1.0000 (0.9823–1.0000)	0.8907 (0.8347–0.9468)
PPV	0.2833 (0.2373–0.3329)	0.4766 (0.5953–0.7003)	0.2956 (0.2441–0.3472)
AUC	0.5943 (0.5728–0.6159)	0.8239 (7976–0.8502)	0.6029 (5613–0.6445)

Se (sensitivity), Sp (specificity), Negative Predictive Value (NPV), Positive Predictive Value (PPV), AUC—Area under the receiver operating characteristic curve.

**Table 5:** ROC Contrast Estimation.

ROC Contrast Estimation and Testing Results by Row						
Contrast	Estimate	SE	95% Wald CLs		Chi-Square	Pr > χ^2^
MST–NRS 2002	0.0086	0.0223	−0.0351	0.0523	0.1489	0.6996
MUST–NRS 2002	0.2296	0.0149	0.2004	0.2587	238.8368	<0.0001
MUST–MST	0.2210	0.0213	0.1791	0.2628	107.2554	<0.0001

**Table 6:** Agreement among the NRS tools based on k-statistics.

Tool	k-Statistics	*p*-Value	Interpret
NRS-2002 vs. MUST	0.2269(0.1805, 0.3133)	<0.0001	Slight
MUST vs. MST	0.4382 (0.3597, 0.5167)	<0.0001	Fair
MST vs. NRS-2002	0.2483 (0.149, 0.3475)	<0.0001	Slight

## Data Availability

Data supporting the findings of this study are included in the article and its Supplementary Materials (tables and graphs). Additional datasets are available from the corresponding author upon reasonable request due to privacy restrictions.

## Supplementary Materials

The following supporting information can be downloaded at: https://www.mdpi.com/article/10.3390/healthcare13233001/s1. File S1.

## Abbreviations

The following abbreviations are used in this manuscript:

BMI	Body Mass Index
KNH	Kenyatta National Hospital
KTRH	Kisii Teaching and Referral Hospital
MST	Malnutrition Screening Tool
MTRH	Moi Teaching and Referral Hospital
MUST	Malnutrition Universal Screening Tool
NPV	Negative Predictive Value
NRS	Nutrition Risk Screening
NRS-2002	Nutrition Risk Screening-2002
PPV	Positive Predictive Value
