# Supplementary material for: Early Malnutrition Risk Detection for Quality Patient Care: An Analysis of Nutrition Risk Screening Tools in Kenyan Referral Hospitals
Source: Healthcare (Basel). 2025 Nov 21;13(23):3001. doi: 10.3390/healthcare13233001 (PMC12692030; doi:10.3390/healthcare13233001)
Supplement: Supplementary file 1 [file healthcare-13-03001-s001.zip › healthcare-3971668-supplementary.pdf]

APPENDICES

1. Informed Consent for Patients

Hello, my name is ______________________________ (name of the research assistant), I am a research assistant in this on behalf of **Mahat Jimale Mohamed,** a PhD student researcher from Kenyatta University.

You have been randomly selected from the Hospitals list of today's/yesterday's admissions to participate in a study about ***Effectiveness and Utilization of Nutrition Risk Screening Tools among Hospitalized Adult Patients in the Kenyan Referral Hospitals.*** About 385 adults newly admitted at the general patients wards of micro-surgical wards at three National Teaching and Referral Hospitals in Kenya (Kenyatta National Hospital, Moi Teaching and Referral Hospital and Kisii Teaching and Referral Hospital) have been randomly selected to participate in this study.

This study aims at identifying the most effective Nutrition Risk Screening (NRS) tool amongst the three validated NRS tools as well as establishing the NRS practices amongst nurses and nutritionists. The findings of this study will be shared and will boost efforts to reduce malnutrition in hospitalized patients for the improvement of clinical outcomes. This study will be conducted only once (i.e., today) for each participant. I therefore kindly request you to consider participating in this study.

If you chose to participate in this study, the following would happen:

1. I will book an appointment with you for a short interview face-to-face at a most appropriate time for you today. The interview will last about 10-15 minutes. The interview will cover questions relating to weight loss, food intake and your disease status. As well, your weight and height measurements will be taken.

2. If you agree to be interviewed, we shall request you to sign a copy of this informed consent form before we proceed. During the interview your identity will be protected and your participation kept confidential by conducting the interview in a private room/section. You will also be identified by the use of codes rather than by your names. The information obtained from you will only be used for purposes of this research and nothing else. Furthermore, after that, all the records will be destroyed by burning the papers and electronic details.

3. Apart from possible concerns about your confidentiality, there are no foreseeable risks or benefits to you for participating in this study and there will be no costs or payments to you. If you have any questions while taking part, you are free to stop me and ask. Although we cannot guarantee absolute anonymity and confidentiality, we will do our best to keep your information anonymous. All data from the questionnaire will be computerised and will be password protected. The questionnaire will be kept in a locked cabinet for safekeeping at Kenyatta University. Your name will not be recorded on the questionnaire. None of your information will be given to any other person in the hospital.

   Please note that your participation in this research is entirely voluntary, and you will not be penalized or lose any benefits due to you from the Hospital on account of your refusal to participate.

For further questions about this research, you may contact Mr. Mahat Mohammed of Kenyatta University on 0722572421. For more information about your rights as a research participant, you may contact the secretary /Chairperson, Kenyatta National Hospital-University of Nairobi Ethics and Research Committee on Telephone No. 2726300 Ext 44102; email uonknh_erc@uonbi.ac.ke

PART 2: GENERAL PRINCIPLES

To the signatory of the consent contained in Part 3 of this document:

You are invited to take part in the research study as described in Part 1 of this informed consent form. It is important that you also read and understand the following general principles, which are applicable to all participants in our research.

1. Participation in the study is completely voluntary and no pressure, however subtle, may be placed on you to take part.

2. It is possible that you may not derive any benefit personally from your participation in the study, although the knowledge that may be gained by means of the study may benefit other persons or communities. In exceptional cases where you do receive personal financial benefits, these are usually for transport to participate and for personal sustenance (e.g. meals) during your participation. You may not be coerced to participate.

3. You are free to withdraw from the study at any time, without stating reasons, and you will in no way be harmed by so doing. However, you are kindly requested not to withdraw from the study without careful consideration, since it may have a detrimental effect on, inter alia, the statistical reliability of the study.

4. By agreeing to take part in the study, you are also giving consent for the data that will be generated to be used by the researchers for scientific purposes as they see fit, with the caveat that it will be confidential and that your name will not be linked to any of the data without your consent.

5. The Ethical Review Committee may request access to information to ensure/inspect the ethical responsibility of practices, in the interest of participants and the public.

6. You will be given access to your own data upon request, unless the Ethics Committee has approved temporary non-disclosure (in the latter case, the reasons in Part 1 will be explained to you).

7. A summary of the nature of the study, the potential risks, factors that may cause you possible inconvenience or discomfort, the benefits that can be expected and the known and/or probable permanent consequences that your participation in the study may have for you as participant, are set out for you in Part 1 hereof.

8. You are encouraged to ask the Study Head or co-workers any questions you may have regarding the study and the related procedures at any stage. They will gladly answer your queries. They will also discuss the study with you in detail.

9. The study objectives are always secondary to your well-being and actions taken will always place your interests above those of the study.

PART 3: CONSENT

Unless you have any questions to ask, may I now request your consent to proceed with the interview.

Participant's Signed Consent

I certify that I have read and understood the purpose and procedure of this study and the implications of my participation. As well, I have received a satisfactory oral explanation and I do hereby freely consent to take part based on adequate information and understanding.

Participant Name ________________________________

Participant's Sign/Thumb print__________________________Date _____________

Declaration by researcher

I have given verbal explanation of the research project procedures and potential risks and /or benefits. I believe that the participant has understood the explanation

Name of researcher _________________________

Sign ___________________________________

Date ________________________________

2. KU Graduate School Research Authorization

KENYATTA UNIVERSITY
GRADUATE SCHOOL

E-mail: dean-graduate@ku.ac.ke
Website: www.ku.ac.ke

P.O. Box 43844, 00100
NAIROBI, KENYA
Tel. 020-8704150

Our Ref: H87/37116/2017 **DATE: 28th October, 2020**

Director General,
National Commission for Science, Technology and Innovation
P.O. Box 30623-00100
NAIROBI

Dear Sir/Madam,

RE: RESEARCH AUTHORIZATION FOR MR. MAHAT JIMALE MOHAMED – REG. NO. H87/37116/17

I write to introduce Mr. Mahat Jimale Mohamed who is a Postgraduate Student of this University. He is registered for Ph.D. degree programme in the **Department of Food, Nutrition & Dietetics.**

Mr. Mahat intends to conduct research for a Ph.D. thesis Proposal entitled, **"Effectiveness and Utilization of Nutrition Risk Screening Tools among Hospitalized Adult Patients in Selected Kenyan Referral Hospitals."**

Any assistance given will be highly appreciated.

Yours faithfully,

PROF. ELISHIBA KIMANI
DEAN, GRADUATE SCHOOL

3. KU-ERC Research Approval

KENYATTA UNIVERSITY
GRADUATE SCHOOL

E-mail: dean-graduate@ku.ac.ke
Website: www.ku.ac.ke

P.O. Box 43844, 00100
NAIROBI, KENYA
Tel. 020-8704150

Internal Memo

FROM: Dean, Graduate School

DATE: 28th October, 2020

TO: **Mr. Mahat Jimale Mohamed**
C/o Department of Food, Nutrition & Dietetics

REF: H87/37116/2017

SUBJECT: **APPROVAL OF RESEARCH PROPOSAL**

===

We acknowledge receipt of your Research Proposal after fulfilling recommendations raised by the Graduate School Board of 1st July, 2020.

You may now proceed with your Data collection, subject to clearance with the Director General, National Commission for Science, Technology & Innovation and Ethics Review Committee, Kenyatta University.

As you embark on your data collection, please note that you will be required to submit to Graduate School completed Supervision Tracking and Progress Report Forms per semester. The forms are available at the University's Website under Graduate School webpage downloads.

Thank you.

JULIA GITU
FOR: DEAN, GRADUATE SCHOOL

CC. Registrar (Academic)
Att. Mr. Richard Chweya

Chairman, Department of Food, Nutrition & Dietetics

Supervisors:

1. Prof. Sophie Ochola
   C/o Department of Food, Nutrition & Dietetics
   Kenyatta University

2. Dr. Judith Munga
   C/o Department of Food, Nutrition & Dietetics
   Kenyatta University

4. NACOSTI Research Approval License

REPUBLIC OF KENYA

NATIONAL COMMISSION FOR SCIENCE,TECHNOLOGY & INNOVATION

Ref No: **622665**

Date of Issue: **18/May/2021**

RESEARCH LICENSE

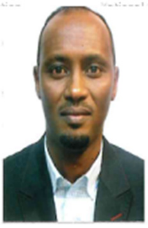

This is to Certify that Mr.. Mahat Jimale Mohamed of Kenyatta University, has been licensed to conduct research in Kisii, Nairobi, Uasin-Gishu on the topic: EFFECTIVENESS AND UTILIZATION OF NUTRITION RISK SCREENING TOOLS AMONG HOSPITALIZED ADULT PATIENTS IN SELECTED KENYAN REFERRAL HOSPITALS for the period ending : 18/May/2022.

License No: **NACOSTI/P/21/10259**

622665

Applicant Identification Number

Director General

NATIONAL COMMISSION FOR SCIENCE,TECHNOLOGY & INNOVATION

Verification QR Code

NOTE: This is a computer generated License. To verify the authenticity of this document, Scan the QR Code using QR scanner application.

5. KNH-UON ERC Research proposal approval

UNIVERSITY OF NAIROBI
FACULTY OF HEALTH SCIENCES
P O BOX 19676 Code 00202
Telegrams: varsity
(254-020) 2726300

KNH-UoN ERC
Email: uonknh_erc@uonbi.ac.ke
Website: http://www.erc.uonbi.ac.ke
Facebook: https://www.facebook.com/uonknh.erc
Twitter: @UONKNH_ERC https://twitter.com/UONKNH_ERC

KENYATTA NATIONAL HOSPITAL
P O BOX 20723 Code 00202
Tel: 726300-9 Ext 44355, 44102
Fax: 725272
Telegrams: MEDSUP, Nairobi

Ref: KNH-ERC/RR/886

7th October, 2021

Mahat Jimale Mohamed
Reg. No.H87/37116/2017
PhD Candidate
Dept. of Food Nutrition and Dietetics
School of Public Health and Applied Human Sciences
Kenyatta University

Dear Mahat,

Research Proposal: Effectiveness and utilization of nutrition risk screening tools among hospitalized adult patients in selected Kenyan Referral Hospitals (P547/07/2021)

This is to acknowledge receipt of your research proposal and to inform you that upon review, the KNH- UoN Ethics and Research Committee made the following observations and suggestions:

Abstract

1. As per the KNH- UoN ERC requirements; provide a Structured Abstract (include relevant subtitles).

Methodology

2. Why is Kenyatta University Teaching Research Referral Hospital not included in the study?
3. Is Kisii Teaching and Referral Hospital "National" or "County"? Is it really a level 6 hospital?
4. Why should the patients be recruited within 24 hours of admission? Does the timing of recruitment with respect to the time of admission affect the credibility and validity of the assessment tools which is the focus for this study?
5. Key Informant Interview:
   i. State clearly when, where this will be done and when, where and by whom the consent will be administered.
   ii. Indicate how the interview will be documented.
6. Will the screening of the patient be a one off exercise? If yes, how will effectiveness of the screening tools be accessed?
7. Logistical and Ethical Considerations:
   i. Include the need for review by the ethics committee of the institutions that are targeted by the study.
   ii. Indicate that authorization will be sought from the facility administrators before commencement of the study.
   iii. Describe measures that will be taken to prevent COVID-19 disease transmission when conducting the study.

Appendices

8. Informed Consent Form: Capture the method to be used for recording the Key Informant Interview and request the participants for their consent.

Protect to discover

Recommendation

Revise and resubmit **three (3)** copies of the full proposal inclusive of the Application Form within a period of **four (4) weeks** with effect from the date of this letter. Include a cover letter that summarizes how you have addressed the comments and note the page number(s) where the changes have been made.

You are also advised to share a soft copy of all the above documents via the ERC email (uonknh_erc@uonbi.ac.ke).

Yours sincerely,

PROF. M.L CHINDIA
SECRETARY, KNH- UoN ERC

c.c. The Dean, Faculty of Health Sciences, UoN
The Senior Director, CS, KNH
The Chair, KNH- UoN ERC
The Chair, Dept. of Food Nutrition and Dietetics, Kenyatta University
Supervisors: Prof. Sophie Ochola, Dept. of Food Nutrition and Dietetics, Kenyatta University
Dr. Judith Munga, Dept. of Food Nutrition and Dietetics, Kenyatta University

6. MTRH Authority to Conduct Research

An ISO 9001:2015 Certified Hospital

MOI TEACHING AND REFERRAL HOSPITAL

Telephone :(+254)053-2033471/2/3/4
Mobile: 722-201277/0722-209795/0734-600461/0734-683361
Fax: 053-2061749
Email: ceo@mtrh.go.ke/directorsofficemtrh@gmail.com

Nandi Road
P.O. Box 3 – 30100
ELDORET, KENYA

Ref: ELD/MTRH/R&P/10/2/V.2/2010

9th May, 2022

Mahat Jimale & Team
Kenyatta University
School of Public Health Sciences
P.O. Box 43844-00100
NAIROBI-KENYA.

ACCEPTABILITY AND RELIABILITY OF NUTRITION RISK SCREENING TOOLS AMONG HOSPITALIZED ADULTS PATIENTS IN THE KENYAN REFERRAL HOSPITALS

You have been authorised to conduct research within the jurisdiction of Moi Teaching and Referral Hospital (MTRH) and its satellites sites. You are required to strictly adhere to the regulations stated below in order to safeguard the safety and well-being of staff, patients and study participants seen at MTRH.

1 The study shall be under Moi Teaching and Referral Hospital regulation.
2 A copy of MTRH/MU-IREC approval shall be a prerequisite to conducting the study.
3 Studies intending to export human bio-specimens must provide a permit from MOH at the recommendation of NACOSTI for each shipment.
4 No data collection will be allowed without an approved consent form(s) to participants unless waiver of written consent has been granted by MTRH/MU-IREC.
5 Take note that **data** collected must be treated with due confidentiality and anonymity.

The continued permission to conduct research shall only be sustained subject to fulfilling all the requirements stated above.

for DR. WILSON K. ARUASA, *MBS, EBS*
CHIEF EXECUTIVE OFFICER
MOI TEACHING AND REFERRAL HOSPITAL

CHIEF EXECUTIVE OFFICER
09 MAY 2022
MOI TEACHING & REFERRAL HOSPITAL

c.c. - Senior Director, Clinical Services
- Director, Nursing Services
- HOD, HRISM

All correspondence should be addressed to the Chief Executive Officer
Visit our Website: www.mtrh.go.ke
TO BE THE LEADING MULTI-SPECIALTY HOSPITAL FOR HEALTHCARE, TRAINING AND RESEARCH IN AFRICA

7. MTRH Institutional Research Ethics Committee *(IREC)* Approval

MTRH/MU-INSTITUTIONAL RESEARCH AND ETHICS COMMITTEE (IREC)

MOI TEACHING AND REFERRAL HOSPITAL
P.O. BOX 3
ELDORET
Tel: 33471//2/3

MOI UNIVERSITY
COLLEGE OF HEALTH SCIENCES
P.O. BOX 4606
ELDORET
Tel: 33471/2/3

Reference: IREC/2021/204
Approval Number: 0004112

4th May, 2022

Mahat Jimale & Team,
Kenyatta University,
School of Public Health Sciences,
P.O. Box 43844-00100,
NAIROBI- KENYA

Dear Mr. Mahat & Team,

ACCEPTABILITY AND RELIABILITY OF NUTRITION RISK SCREENING TOOLS AMONG HOSPITALIZED ADULT PATIENTS IN THE KENYAN REFERRAL HOSPITALS

This is to inform you that ***MTRH/MU-IREC*** has reviewed and approved the above referenced research proposal. Your application approval number is **FAN: 0004112.** The approval period is **4th May, 2022- 3rd May, 2023.**
This approval is subject to compliance with the following requirements;

i. Only approved documents including (informed consents, study instruments, Material Transfer Agreements (MTA) will be used.
ii. All changes including (amendments, deviations, and violations) are submitted for review and approval by ***MTRH/MU-IREC***.
iii. Death and life threatening problems and serious adverse events or unexpected adverse events whether related or unrelated to the study must be reported to ***MTRH/MU-IREC*** within 72 hours of notification.
iv. Any changes, anticipated or otherwise that may increase the risks or affected safety or welfare of study participants and others or affect the integrity of the research must be reported to ***MTRH/MU-IREC*** within 72 hours.
v. Clearance for export of biological specimens must be obtained from ***MOH at the recommendation of NACOSTI*** for each batch of shipment.
vi. Submission of a request for renewal of approval at least 60 days prior to expiry of the approval period. Attach a comprehensive progress report to support the renewal.
vii. Submission of an executive summary report within 90 days upon completion of the study to ***MTRH/ MU-IREC.***

Prior to commencing your study; you will be required to obtain a research license from the National Commission for Science, Technology and Innovation (NACOSTI) https://oris.nacosti.go.ke and other relevant clearances from study sites including a written approval from the CEO-MTRH which is mandatory for studies to be undertaken within the jurisdiction of Moi Teaching & Referral Hospital (MTRH) and its satellites sites.

INSTITUTIONAL RESEARCH & ETHICS COMMITTEE
04 MAY 2022
APPROVED
P. O. Box 4606 - 30100 ELDORET

Sincerely,

PROF. E. WERE
CHAIRMAN
INSTITUTIONAL RESEARCH AND ETHICS COMMITTEE

cc	CEO	-	MTRH	Dean	-	SOP	Dean	-	SOM
	Principal	-	CHS	Dean	-	SON	Dean	-	SOD

8. KTRH Institutional Research Ethics Committee *(IREC)* authority

KISII COUNTY GOVERNMENT
DEPARTMENT OF HEALTH

Telegramme "Medical"
Telephone: (058) 31310 Kisii
E-Mail kisiihospital@gmail.com
Web: www.kisiihospital.org.ke
***Ref:* ISERC/KTRH/002/22**

CHIEF EXECUTIVE OFFICER
KISII TEACHING & REFERRAL HOSPITAL
P.O Box 92 – 40200,
KISII
Date: 1st MARCH 2022

TO WHOM IT MAY CONCERN

RE: AUTHORIZATION OF MAHAT JIMALE MOHAMED TO CONDUCT A RESEARCH STUDY TITLED 'EFFECTIVENESS AND UTLIZATION OF NUTRITION RISK SCREENING TOOLS AMONG HOSPITALIZED ADULT PATIENTS IN KENYAN REFERRAL HOSPITALS '

The above subject matter refers.

Having met all the requirements, **MAHAT JIMALE MOHAMED,** from **KENYATTA UNIVERSITY REG. NO. H87/37116/17** is authorized to conduct research in the Kisii Teaching and Referral Hospital within the next 365 days from the date of this letter.

The study will be carried out subject to adherence to the laid down procedures. The researcher should observe confidentiality of study subjects and any collected samples at all times. He should submit the final report to the County Research Unit for retention and use.

Study timelines exceeding 365 days will require a renewal of approval application that will include an annual progress report.

Kindly accord him any support that he requires that falls within the scope of this study.

Yours faithfully,

The Head Research
Quality Assurance & Standards
KISII TEACHING & REFERRAL HOSPITAL
P. O. Box 92 - 40200, Kisii

DR. DANIEL MUVENGEI
COUNTY RESEARCH OFFICER
FOR: CHIEF EXECUTIVE OFFICER
KISII TEACHING AND REFERRAL HOSPITAL

Cc: -
DD – CLINICAL SERVICES
